# Supplementary material for: Effect of 3-D depth structure, element size, and area containing elements on total-element overestimation phenomenon
Source: PLoS One. 2024 Feb 27;19(2):e0299307. doi: 10.1371/journal.pone.0299307 (PMC10898729; doi:10.1371/journal.pone.0299307)
Supplement: Attachment — Submitted filename: Response_to_reviewer1-2.pdf [file pone.0299307.s002.pdf]

The following are our responses to Reviewer #1's specific comments:

Reviewer #1: Nice revision. There is one thing I don't understand -- when the element number of the standard stimulus was 100, the element range of the comparison was 70-130 (ER= -0.3-0.3, line 175-178). In theory, the PSEs must be within the range of -0.3-0.3, right? Why are some participants' PSEs over 0.3 in Fig 3-6 (some even higher than 0.5)? Their data should be removed if they are artifacts.

We sincerely appreciate your careful reading of our manuscript and apologize for not mentioning the critical information on the method used to calculate the PSE. We have added detailed information on the PSE calculation method to the revised manuscript. We hope that the added information clarifies the your concerns.

We calculated the coefficient of determination (R^2) for each condition and observer in Experiments 1 to 4. When the value of R^2 did not satisfy our criterion, we asked the observer to repeat the same condition three times on different days, if available. The incremental step size (ER step size) or number of elements in the comparison stimulus was changed to obtain the R^2 value over the criterion. We used an ER step size of 0.1 on the first and second days and 0.2 on the third. When the observers' data did not meet the criterion, we changed the number of elements in the comparison on the second and third days; the smallest number could differ among observers. The experimenter determined the smallest number based on observing the first or second day's data on the second or third day, respectively; some observers' PSE exceeded 0.3. Accordingly, the PSE values over 0.3 shown in Figures 3–6 are not artifacts.

Other minor suggestions:

line 31: "The two stimuli were presented side-by-side, with similar diameters" -- Similar or the same/identical diameter?

This was a mistake. The word "similar" should be replaced with "the same." This has been corrected. Thank you for the comment.

line 33: "the overestimation phenomenon occurred for the stimuli we used" -- Which stimuli? The three-dimensional standard stimuli?

Yes. We replaced "the stimuli" with "the three-dimensional standard stimuli."

line 116: The participants are "patients"?

Thank you for the comment. We corrected the typo.

Fig 4, 5, 6: It would be better to have different colors for individual data coding. It's hard to see the trend when some points overlap.

We revised Figures 4, 5, and 6 as suggested.

It would be great to provide the density information of the stimuli for each experiment (element/deg2) so that the readers can compare the results with other studies in numerosity.

Thank you for this suggestion. We have added the density information to the Stimulus section of each experiment.

Reviewer #2: The authors have addressed most of my queries from the first round of reviews. The manuscript is much improved and easier to follow. I thank the authors for taking care in addressing these issues and making extensive and appropriate changes. I have a few remaining concerns that should be addressed:

*1. Interference theory: I still don't get this proposal. Sure, one can *assume* that disparity processing interferes with numerosity processing. But that is simply ad-hoc based on the data. Any set of findings can similarly be accommodated by making appropriate assumptions. This would make the theory unfalsifiable. This is exemplified in the paper itself, which adds ad-hoc 'assumptions' as and when needed. For example, lines 693-694 add the assumption that area also affects the disparity-numerosity interference! Of course, if you assume this, you can explain the data. But what is the basis of such as assumption? One needs independent reasons to suggest that, not just the observed data. The latter makes it unfalsifiable.*

We agree that assumptions without any independent reasons should be avoided. Accordingly, we have revised the paragraph, including lines 766–767 and what follows. In the revised version, we clearly state that our argument is based on our own speculation.

Further, why should there be any interference between ostensibly unconnected processes (disparity and numerosity mechanisms)?

In the literature, considerable research has found that disparity processing interacts with processing visual primary properties such as color (e.g., Yang & Shevell, 2002), motion (e.g., Anstis & Harris, 1974), spatial frequency (e.g., Cobo-Lewis & Yei-Yu, 1994), and density (e.g., Sun & Baker, 2018).

If numerosity is an independent primary visual property, as Burr and Ross (2008) claims, it is unlikely that disparity and numerosity processing are ostensibly unconnected. Accordingly, we speculate that there may be interaction or 'interference' between disparity and numerosity processing (please see our next response for our intention to use the term 'interference'). We discuss the idea that numerosity processing can interact with disparity processing in the General Discussion section.

*Second, why should such an interference cause *overestimation* rather than underestimation? Seems very convenient to assume interference, which in turn only leads to overestimation.*

We apologize for the confusion. We did not intend to use the term 'interference' to refer only to the overestimation phenomenon of the element number. Aida et al. (2020) proposed "a hypothesis that holds that processing the disparity of 3-D stimulus elements puts a load on the numerosity estimation …" (p. 20), although they did not call the hypothesis a "disparity-processing interference" hypothesis. We interpret the sentence as meaning that the load would disturb or interfere with the normal functioning of the numerosity estimation process and may affect the accuracy or precision of numerosity estimation. The interference can lead to over- or under-estimation and more or less precise estimations of the 3-D stimulus compared to the 2-D stimulus. Thus, interference does not automatically refer to overestimation. Empirical evidence has shown that the number of 3-D elements is overestimated (Aida et al., 2015, 2020; Schütz, 2012), and 3-D numerosity estimation is as precise as that of 2-D (Aida et al., 2020). We explain our usage of interference in the revised General Discussion section.

2. *A related concern arises for other 'assumptions' made to explain some findings: that interference from contrast-based disparity mechanism increases with area size. What is the basis for such an assumption? If it is the data, then that is circular. There should be independent reasons to suggest such mechanisms.*

We appreciate this suggestion. In the revised General Discussion section, we have stated that our discussion on the area effect of a 3-D stimulus is based on speculation. The speculation is derived from the proposal that the contrast-based disparity mechanism depends on the disparity stimulus size (Wilcox & Allison, 2009) and the observation that the observer can utilize the contrast-based motion information to estimate the numerosity (Kramer et al., 2011).

a. *Why does interference not increase beyond the intermediate area, if it were the case that area*

increases interference?

Thank you for the comment. This is an interesting question, but unfortunately we do not yet have a clear answer. We have mentioned the issue in the revised General Discussion section.

3. There are several offhand claims that don't seem to be supported by the data:

a. Lines 425-426: overestimation occurs when element size is large. Huh? The data seem to show that element size has no effect (the ratio is the same for all sizes). What is this conclusion based on? Is it effect sizes and p-values, which are notoriously noisy (especially with such small sample sizes)? Seems not supported.

We apologize for the confusion. In this paragraph and the next, we have attempted to explain why the overestimation phenomenon occurred in this study and previous studies (Aida et al., 2015, 2020; Schütz, 2012) but not in Bell et al. (2015). In the revised manuscript, we have deleted the first paragraph and revised the next paragraph to better clarify our point.

b. The above is then discussed as if that is a clear finding (paragraph starting on line 431) that needs an explanation. There is no evidence that overestimation depends on a difference in element size.

We agree that there is no evidence that the size of elements affects the overestimation phenomenon. As mentioned above, we have deleted the statements that could cause confusion.

c. Lines 515-516 (and other places): overestimation increases with area (noted in point #2 above). Be careful with this claim. It seems like it is being interpreted as if there is a continuous increase in overestimation with increasing area, which is definitely not the case. There is more overestimation from small to medium area, but not beyond that (at numerosities 100 and 150). If there is an effect of area, it is restricted to small-medium area range, not beyond. This should be acknowledged here and in the General Discussion.

We appreciate the comment. We have clearly stated that the effect of area is restricted to the small-medium area range in the revised Results and Discussion section in Experiment 4 and the General Discussion section.

d. Lines 699-700: It is stated as if the interference theory is correct. I didn't see any evidence that convincingly points to this. In fact, without additional 'assumptions' it cannot account for current

findings. Please tone down the claims.

As suggested, we revised the statement and toned down the claim.

4. *Control experiment: I am happy to see that the authors conducted a control experiment in line with the other reviewer's suggestion. However, this control cannot distinguish the effect of 'novelty' or a response 'bias' towards the 3-D stimulus. To achieve that, the standard should be of a different color (say white) or shape (say triangles). Otherwise, it is unclear if a different looking stimulus did not bias responses. I am aware of the authors' arguments for why it might not be a bias, but a direct demonstration is worth a thousand such arguments. A control experiment would clearly put such concerns to rest.*

Thank you for the comment. We did not understand the difference between "the effect of novelty and a response bias toward the 3-D stimulus." If the effect of novelty implies that observers detect the non-zero binocular disparity of the 3-D stimulus, recognize it as "novel," and tend to select it, we think it is the observers' response bias. The reviewer suggested using the standard 3-D stimulus containing, for example, white elements to distinguish "the effect and a response bias." According to the suggestion, the comparison 2-D stimulus should be black elements whose size and color are the same as those of standard elements. If we observed the overestimation phenomenon in this "control" experiment, the result would indicate that the phenomenon can occur under a more generalized condition. However, this result alone does not exclude the possibility that the result stems from the 3-D bias. If we did not observe the phenomenon, it would show that the phenomenon occurs when the 2-D and 3-D stimuli have the same color or that the difference in element color affects the phenomenon. At present, we do not comprehend the logic that the control experiment suggested can discriminate the effect of novelty and a response bias towards the 3-D stimulus. Ultimately, we decided not to perform the control experiment suggested at this time until we achieved further clarity. Furthermore, we have deleted the previous description of control experiments.

We agree that it is interesting to know whether the color difference or the novelty of a stimulus affects the numerosity judgment, and the question is worth addressing in future studies.

5. Minor concerns:

a. Abstract: The final sentence is not a conclusion. Please state the take-away message of the study (even if it is not conclusive). Right now, it is weak, vague, and uninformative.

We rewrote the sentence. Please see the revised Abstract.

b. Are there any predictions for the 3 possible hypotheses for the manipulations undertaken in this study? [Intro: para starting line 94].

There is no prediction for the hypothesis examined in this study. We have stated this point clearly in the revised manuscript.

c. Out of curiosity, what is the perceived orientation of the cylinder (seven-POTS stimulus) in Experiment 1?

Observers perceived a cylindrical cluster of elements perpendicular to the front parallel plane. We have described the percept in the revised manuscript.

d. Thanks for the clear plots. I second the other reviewer's suggestion of plotting a psychometric curve. I would additionally like to see the average of all participants plotted (proportion of 'selected comparison stimulus' as a function of 'elements ratio') along with error bars and a psychometric curve fitted to these average data for each experiment.

We apologize that our description of the data analysis was inaccurate and misleading. The step size of the element ratio or the number of elements in the comparison stimulus differed among observers when the criterion for the PSE ratio was met, as mentioned in response to Reviewer 1's comment. Thus, plotting the average PSE ratio as a function of the element ratio is very difficult.

e. Correction for multiple comparisons: while such a correction (Holm) has been applied to some comparisons, it has not been applied to all. For example, the one-sample t-tests against zero have not been corrected, and they should be.

We appreciate this comment. We have calculated the corrected t-test values against zero for every sub-condition in each condition.

f. Line 309 mentions an ANOVA. What ANOVA? So far, only t-tests have been conducted.

Thank you for pointing this out. "ANOVA" should be "t-test. We have corrected it in the revised manuscript.

g. Experiment 4 intro: What's expected with this manipulation and why? Is there any reason to think that different numerosities will display different patterns of results? If so, why?

We examined the area effect under a more general condition because we were interested in whether the effect operated differently for 3-D and 2-D stimuli. To reveal the effect of 3-D stimuli under more generalized conditions would enhance our understanding of the mechanism of numerosity estimation. We have discussed this issue in the preamble of Experiment 4 and the Result and Discussion sections in Experiments 3 and 4.

6. Language, clarity, and typos (in order of appearance in the manuscript, not importance)

a. Line 42: Please rephrase for clarity.

b. Line 44, 453, 454, and several other places, please consider dropping the word 'size' in relation to area (such as in 'area size'). Area is already a measurement, there is no need to say size. It is like saying length size or radius size. It is redundant.

c. Line 57: Delete 'significant' (it is neither a statistical test nor a qualitative judgment).

d. Lines 73-76: Needs to be phrased better, or more details are needed. It is unclear at the moment.

e. Lines 91: 'other factor(s) rather than' should be 'factor(s) other than'

f. Line 99, 280: Replace the semicolon (;) with a colon (:).

g. Lines 164-165: Not clear; please rephrase

h. Line 181: Replace 'the' with 'a'

i. Line 211: Should 'the' be 'three'?

j. Line 262: Perhaps this phrasing would be better: "and among the remaining 30, 15 elements had +5.2 or +7.8 arcmin crossed disparity each"?

k. Line 273: 'before the numerosity discrimination task' is duplicated.

l. Line 428: Replace 'more significant' with 'larger' (as above in 6c). Similarly, line 521 should have 'higher' instead of 'more significant'. Please consider changing similar phrasing elsewhere.

m. Line 648: 'interacting' should be 'interact'

n. Line 658: What is 'two-PODS'?

o. Line 670: Delete 'between'

We corrected our typos and revised the incorrect or ambiguous expressions as suggested. Otherwise, we deleted or changed the expressions or words listed.

p.s. I understand the argument about power, and that viewing disparity images can be tiring to the eyes, but I am disappointed that this is given as a reason for not collecting more trials/participants. In visual psychophysics, in our lab, we routinely collect data for 2-3 hours per participant on a task

(sometimes with mirror stereoscopes), often on 20 participants in a given experiment. Given that the current experiment is only 10-15 minutes long, I am surprised that with sufficient breaks, more data were not collected (three sessions separated by 10 min each would still be less than an hour! And would triple the data collected). It goes without saying that reducing noise in measurements is imperative for good science.

We agree that researchers should collect a sufficient number of trials/participants for an experiment. We would like to explain why we adopted the procedure used in this study. The procedure was based on our observation in preliminary experiments, which suggested that some observers responded rather sloppily when they responded successively for more than 20 minutes. It seemed that they lost the motivation to participate in the experiment. Therefore, we had a break of more than a week between one session and the next, hoping that the break would reinvigorate the observers' motivation. The reviewer might be surprised as to why the students behaved in such a way. We speculate that it may be because our observers were not psychology students. Thus, they may have lost their curiosity about psychophysical experiments more easily than psychology students would in the same situation.
