# Supplementary material for: Effect of 3-D depth structure, element size, and area containing elements on total-element overestimation phenomenon
Source: PLoS One. 2024 Feb 27;19(2):e0299307. doi: 10.1371/journal.pone.0299307 (PMC10898729; doi:10.1371/journal.pone.0299307)
Supplement: Attachment — Submitted filename: Response_to_reviewer_2.docx [file pone.0299307.s003.docx]

The following are our responses to Reviewer #1’s specific comments:

*Reviewer #1: In the last revision the authors wrote in their response letter that they conducted a baseline experiment for Experiment 1 -- 'The result of the baseline experiment showed that when 2-D stimuli were used as the standard and comparison, there was no numerosity overestimation for the standard stimulus'. Somehow I can't find the baseline experiment in the current manuscript -- it would be very helpful to include it in the paper. Other major questions are well answered.*

We apologize for not including our discussion regarding the baseline in the previous version of the manuscript. As requested, we have included this discussion in the revised manuscript. Please note that the baseline or control experiment was newly conducted.

*Reviewer #2: The authors have addressed almost all of my concerns. The manuscript is streamlined and clear. I have a few outstanding minor queries and suggestions that I hope the authors will take on board.
1. Experiment 2-4: the various combinations (3 main-variable manipulations x 7 numerosities x 2 standard locations x 10 repetitions)*

*should lead to 420 trials, but is stated as 210. Is that a typo or were the locations not balanced (but were randomly chosen instead)?*

We appreciate this comment. We decreased the trial number through Experiments 2-4, as compared with Experiment 1, in order to shorten the total time required to complete each experiment. The number of trials was 210, but the number of repetitions was 5—not 10.

*2. A central motivating factor for the study has been the discrepancy between earlier results (Bell et al. and Aida et al. – in relation to observing overestimation in a 3-D volume). However, this discrepancy is not resolved by the current study, since element size does not seem to matter, and in the current experiments, overestimation is observed at all tested numerosities and areas. The authors speculate that the difference might lie in duration (line 439 – 455). Obviously testing it directly would be the ideal way forward. But if that cannot be done, please move this paragraph and discuss it in the General Discussion, rather than tuck it away in the results of Experiment 2.*

We agree with this comment. We have moved our discussion on the discrepancy between the results of Bell et al. and Aida et al. to the General Discussion.

*3. My apologies for not being clearer in my earlier comment about the ‘control’ experiment. I was merely pointing out the inadequacy of the previous ‘baseline’ experiment to rule out response bias – I did not mean to confuse novelty and response bias. To really rule out response bias, the ‘standard’ 2-D stimulus should have been made distinct in some way. If you still find overestimation for the standard, it means that participants are biased towards an ‘interesting’ or ‘novel’ stimulus. Given that all 3-D stimulus in this study lead to overestimation, it is important to rule out such a bias towards 3-D stimulus. Yes, the authors make a case for why response bias might not be plausible here, but I don’t find it fully persuasive (see below).*

*a. The evidence for why response bias might have occurred can also be explained by response bias: The effect of disparity (stepwise 3-D) on overestimation can be explained by considering that the more depth that is observed, the more response bias there might be towards it. And perhaps it saturates at some point, hence there might not be much difference between clearly 3-D structures such as 2-POTS and 3-POTS with comparable disparities. In the current study, all manipulations lead to overestimation, indicating that such a bias cannot be ruled out. My suggestion was to do an experiment,* *with the same procedure, but instead of a 3-D structure, merely change the attributes of the standard stimulus elements. Thus, if participants have a bias towards a novel/interesting stimulus, they should pick that one too.*

As per the reviewer’s suggestion, we conducted a control experiment by modifying one of the attributes of a standard stimulus. We maintained the same procedure and other stimulus characteristics in the control experiment as those in the main experiments. In this experiment, the standard and the comparison were 2-D stimuli that differed only in the color or shape of their elements. Specifically, the standard stimulus had triangular black or rectangular red elements, whereas the comparison stimulus had rectangular and black elements. The findings of the control experiment did not indicate the overestimation phenomenon. The result suggests that observers did not usually opt for a standard stimulus compared to the comparison stimulus.

*4. Line 785-787: Can the authors elaborate on why the disparity-processing model cannot account for the results of Experiments 3 and 4?*
We thank you for the comment. We have added our discussion regarding why the model cannot explain the results.

*There still remain several typos and grammatical errors. Please go through carefully and fix them. Here, I point out a couple that might make a substantial difference in interpretation:
1. Abstract: Line 35 should be: ‘while the number of overlapping stereo surfaces.*

*2. Line 604: 195 x alpha might be missing a term: all the others are in the form of a+b*alpha.*

We thank you for your careful reading. We have made the necessary corrections as suggested and have tried to fix our careless writing. We hope that we have dealt with all our typos.

We hope you and the other two referees will find this revised version suitable for publication in PLOS ONE.

Yours sincerely,

Yusuke Matsuda

Assistant Professor

Suwa University of Science

Department of applied Information Engineering

Faculty of Engineering
