# Supplementary material for: Effect of 3-D depth structure, element size, and area containing elements on total-element overestimation phenomenon
Source: PLoS One. 2024 Feb 27;19(2):e0299307. doi: 10.1371/journal.pone.0299307 (PMC10898729; doi:10.1371/journal.pone.0299307)
Supplement: Attachment — Submitted filename: Response_to_reviewer_3.docx [file pone.0299307.s004.docx]

The following are our responses to Reviewer #2’s specific comments:

*Reviewer #2: Thank you for conducting the control experiment. The results are convincing that there is no response bias towards one or the other stimulus. All my remaining concerns have been addressed.
One final thing. Thanks for clarifying that there are 5 repetitions per location in Experiments 2-4. However, in Experiments 3 and 4, it is still noted as 10 repetitions. See lines 521-522 ["there were 210 trials (three areas × seven numbers of elements × two locations of the standard × ten repetitions)"] and 642-644 [" there were 210 trials (three sizes of areas × seven numbers of elements × two locations of the standard × ten repetitions) for each observer. This is *mathematically impossible*. If you include two locations, then please change # of repetitions to 5, or omit locations and retain 10 repetitions. Otherwise, you get 420 trials (3x7x2x10 = 420).*

We apologize for the misleading revisions in our previous manuscript. In Experiments 1–4, the position of the standard stimulus was determined randomly in each trial rather than five times in each position. Therefore, there were a total of 210 trials in Experiments 3 and 4. The following text has been revised:

Line 297 – 299,

The placement of the 3-D standard stimulus (to the left or right of the midsagittal plane for each trial and observer) was randomly determined.

Line 427 – 428,

The location of the 3-D standard was randomly determined, as in Experiment 1.

Line 521,

We removed the phrase “two locations of the standard.”

Line 521 – 522,

The location of the 3-D standard was randomly determined, as in Experiments 1 and 2.

Line 642,

We removed the phrase “two locations of the standard.”

Line 640 – 643,

The location of the 3-D standard was randomly determined using the same method as in Experiments 1–3. Similar to Experiments 1–3, the observer may have performed each session three times.

In addition, we revised grammatical problems, repeated expressions, typos, and other issues.

Line 239 – 242,

If the number of standard stimulus elements is 100 (which is the same as in Experiment 1), a PSE of 0.09 indicates that “the 100 elements of the standard stimulus and the 109 elements of the comparison stimulus are perceived as the same number.”

Line 271 – 272,

The distance between the center of the standard and comparison stimuli was 13.8 arc deg.

Line 414 – 415,

and the center-to-center distance between the standard and comparison stimuli was 13.8 arc deg.

Line 501 – 503,

The center-to-center distance between the standard and comparison stimuli was the same for all three size conditions, i.e., was 13.8 arc deg, as in Experiments 1 and 2.

Line 610 – 611,

The center-to-center distance between the standard and comparison stimuli was 13.8 arc deg in all three size conditions, as in Experiment 3.

Line 661

areas for the 50-element standard (adj.*p*s > .05).

Other minor revised are highlighted in the manuscript.

We hope that you will find this revised version suitable for publication in PLOS ONE.

Yours sincerely,

Yusuke Matsuda

Assistant Professor

Suwa University of Science

Department of applied Information Engineering

Faculty of Engineering
